# The radiological diagnosis of pneumonia in children

**DOI:** 10.15172/pneu.2014.5/482

**Published:** 2014-12-01

**Authors:** Kerry-Ann F. O’Grady, Paul J. Torzillo, Kieran Frawley, Anne B. Chang

**Affiliations:** 16 Queensland Children’s Medical Research Institute, Queensland University of Technology, Level 4, Foundation Building, Herston, Queensland Australia; 26 Sydney Medical School, The University of Sydney, Camperdown, Sydney, Australia; 66 Departments of Respiratory Medicine and Intensive Care Royal Prince Alfred Hospital, Camperdown, Sydney, Australia; 36 Department of Radiology, Royal Children’s Hospital, Brisbane, Queensland Australia; 46 Child Health Division, Menzies School of Health Research, Charles Darwin University, Tiwi, Northern Territory Australia; 56 Queensland Children’s Respiratory Centre, Royal Children’s Hospital, Brisbane, Queensland Australia

**Keywords:** radiology, diagnosis, pneumonia, children, utility

## Abstract

Despite the importance of paediatric pneumonia as a cause of short and long-term morbidity and mortality worldwide, a reliable gold standard for its diagnosis remains elusive. The utility of clinical, microbiological and radiological diagnostic approaches varies widely within and between populations and is heavily dependent on the expertise and resources available in various settings. Here we review the role of radiology in the diagnosis of paediatric pneumonia. Chest radiographs (CXRs) are the most widely employed test, however, they are not indicated in ambulatory settings, cannot distinguish between viral and bacterial infections and have a limited role in the ongoing management of disease. A standardised definition of alveolar pneumonia on a CXR exists for epidemiological studies targeting bacterial pneumonias but it should not be extrapolated to clinical settings. Radiography, computed tomography and to a lesser extent ultrasonography and magnetic resonance imaging play an important role in complicated pneumonias but there are limitations that preclude their use as routine diagnostic tools. Large population-based studies are needed in different populations to address many of the knowledge gaps in the radiological diagnosis of pneumonia in children, however, the feasibility of such studies is an important barrier.

## 1. Introduction

Pneumonia remains the most important cause of mortality and morbidity in young children globally [1,2]. In addition to the impact of acute disease, respiratory infections (especially when repeated) in young children are associated with long-term lung function abnormality and disease in adults [3]. Early diagnosis and management are critical to short- and long-term health outcomes with several clinical guidelines now available for both developing and developed country settings, albeit with concerns about the inconsistencies between these documents [4]. The implementation and effectiveness of the guidelines vary widely within and between countries and, in many regions, improvements are still required in the diagnosis and management of pneumonia at the community level [5–9].

Despite the commonality of pneumonia in children, disagreement remains about diagnosis in both clinical and research settings [9,10]. Many factors contribute to these differences, including: health systems resourcing, the number of possible causative micro-organisms, host and environmental factors, timing of presentation to a health service, expertise of the health service providers at various levels of the health care system, availability of diagnostic facilities and the absence of a true diagnostic gold standard [11,12]. The World Health Organization (WHO) clinical definition developed for the community setting in developing countries is based on the presence of cough and tachypnoea [13]. This definition was developed particularly with the intention of identifying children who had bacterial pneumonia and required antibiotics [14]. However, while highly sensitive, this definition lacks specificity. The major reason for this is the problem of viral infections affecting airways but not lung parenchyma in children with these infections [15], although many of these children may have co-infection particularly with *Streptococcus pneumoniae* [16]. In addition, in settings where there is a high prevalence of conditions with similar symptoms and signs like malaria and tuberculosis (TB), differentiating pneumonia from malaria [17,18] and TB (with human immunodeficiency virus) at the time of presentation may be difficult [18,19]. Pneumonia may also be masked in cases of severe diarrhoea and hypokalaemia [20].

In the appropriate setting (e.g. trained health care professionals and diagnostic services), other factors may be considered to improve the specificity of the diagnosis of pneumonia. These factors include clinical symptoms and signs (e.g. crackles) and objective tests (e.g. pulse oximetry and radiology). The microbiological cause is often considered diagnostic but there are many limitations to this assessment. Despite advances in identifying microorganisms using highly sensitive molecular techniques, ascribing causation is problematic [11]. Even when the same molecular detection techniques for viruses are used, the site of specimen collection influences results [21]. The ideal samples for determining aetiologic agents in bacterial pneumonia are lower airway specimens. It is usually neither necessary nor feasible to obtain either bronchoalveolar lavage (BAL) or needle lung aspirate specimens in acute pneumonia [11]. Thus, it is not surprising that the chest radiograph (CXR) has long been considered the ‘gold standard for the diagnosis of pneumonia in children’. Historically, this has been largely driven by the need to identify bacterial pneumonia and hence inform the use and choice of antibiotic therapy [9].

Here, we review the role of radiology in paediatric pneumonia. We predominantly discuss the use of the CXR for clinical and research purposes. We also review other diagnostic methods including lung ultrasonography (LUS) and briefly discuss computed tomography (CT) and magnetic resonance imaging (MRI).

## 2. Chest Radiograph (CXR)

Although, the CXR is the most widely used diagnostic imaging tool for paediatric pneumonia, its use in the clinical context is controversial with recent guidelines advocating that CXRs for the diagnosis of pneumonia in the community setting are unwarranted [22,23] (further discussed below). Nevertheless, here we review the various aspects of CXRs related to the diagnosis of childhood pneumonia.

### 2.1 Patterns of CXR abnormalities

There is a spectrum of radiological appearances that are consistent with the clinical and pathological diagnosis of pneumonia, ranging from complicated pneumonia (e.g. pneumonia with empyema and necrotising pneumonia), simple or uncomplicated pneumonia (e.g. lobar consolidation) to mild interstitial changes [24]. The characteristics of childhood pneumonia on CXRs generally assume a pattern approach based on pathologic and radiologic characteristics [25].

**Figure 1:**
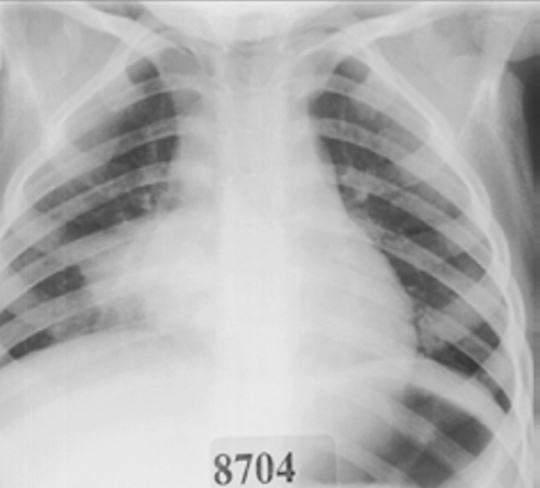
Chest radiograph showing right lower lobe pneumonia

Lobar pneumonia is usually considered to be associated with specific bacterial infections such as *Haemophilus influenzae* type b (Hib), *S. pneumoniae* and *Klebsiella pneumoniae* [25,26]. Features on CXRs are a non-segmental, homogenous consolidation predominantly involving one lobe with air bronchograms (large bronchi remain patent and air-filled in contrast to the adjacent non-aerated lung) [25] (Figure 1). Multilobar pneumonia can occur with a number of different bacteria and is associated with more severe disease [27,28].

Bronchopneumonia is thought to be usually associated with infections due to gram-negative bacteria, *Staphylococcus aureus* and some fungi [25]. The radiological appearance of bronchopneumonia varies depending on the severity of disease. Mild disease can manifest as peribronchial thickening and poorly defined air-space opacities; inhomogeneous patchy areas of consolidation involving several lobes reflect more severe disease. When confluent, bronchopneumonia may resemble lobar pneumonia [25].

**Figure 2:**
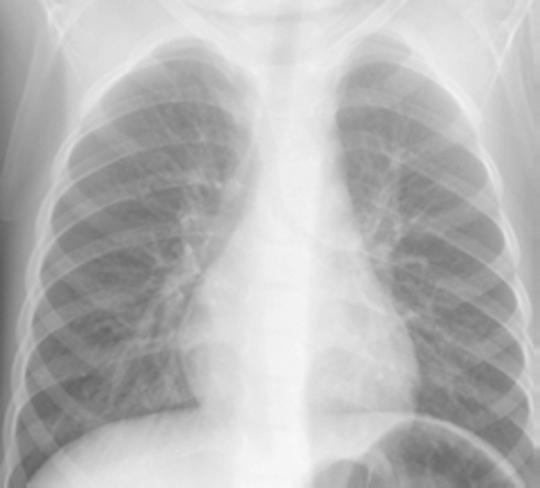
Chest radiograph showing interstitial infiltrates

Interstitial pneumonia (Figure 2) is typically associated with viral infections such as influenza virus and respiratory syncytial virus (RSV) [29–31]. Few pathogens are associated with characteristic CXR abnormalities. *Pneumocystis jirovecii* is characterised by oedema and cellular infiltrates predominantly involving the interstitial tissue of the alveolar septa and surrounding small airways and vessels [25]. A reticular or reticulonodular pattern and septal lines (Kerley B lines) may be seen in *P. jirovecii* infections [32]. Ground glass opacities and multifocal consolidation are associated with severe disease and have been commonly observed in children with severe pandemic influenza infections [33,34].

Lung complications of pneumonia include empyema and, less commonly, pulmonary abscess and necrotising lung. Empyema is defined as intrapleural pus or a moderate to large exudative parapneumonic effusion which can progress to being loculated, with further development of a fibrinous peel [35]. The CXR cannot diagnose empyema, only the presence of parapneumonic fluid [36]. Loculated effusions may be difficult to distinguish from a peripheral lung abscess [36]. Lung abscesses manifest radiologically as cavities that may be isolated or occur within areas of consolidation [25]. Necrotising pneumonias initially appear as small lucencies within an area of consolidation, progressing to larger, fluid filled cavities [25]. The CXR will reveal the presence of larger cavities and abscesses, although minor changes may only be visible on CT [37]. In complicated pneumonia, CTs will reveal abnormalities not detected on CXRs [38].

### 2.2 Limitations of CXR patterns

Other than for complicated pneumonia, there are limitations to this pattern approach, particularly at an individual level in the clinical setting where host factors such as age, comorbidities and immunologic status can modify the radiologic manifestations of pneumonia. In some settings there is wide variation in the use of CXRs in emergency departments (EDs) but no corresponding association with the proportion of children diagnosed with pneumonia [39]. The utility of clinical signs and symptoms present at the time of CXR to predict a radiological diagnosis of pneumonia, particularly in non-severe cases, varies across studies [18,40–45]. In addition, the interpretation of CXR findings is dependent on the quality of the film and the expertise of the reader, with several studies demonstrating varying degrees of concordance between clinicians [46–49], between clinicians and radiologists [48,50,51] and between radiologists [44,52]. Despite the frequency of use of CXRs, there is limited evidence to support its routine use in distinguishing between viral and bacterial infections and its ongoing use in clinical management once a diagnosis of pneumonia has been made.

### 2.3 Distinguishing between viral and bacterial infections

Differentiating between viral and bacterial pneumonia continues to be a major clinical challenge, whether it be based on clinical findings, diagnostic tests or both [12,53]. Comparable, well-designed studies that have used credible reference standards such as lung aspiration or comprehensive panels of laboratory investigations to establish the viral or bacterial origin of radiologically diagnosed pneumonia are limited in number. This is further compounded by the increasing description of multi-organism infection or detection for a single clinical episode with the use of molecular diagnostic methods such as polymerase chain reaction. Increasingly, viral-viral, viral-bacterial and bacterial-bacterial interactions in the pathogenesis of respiratory infections are recognised with *in-vitro* and *in-vivo* animal [54] and human studies [55,56]. Thus, although viruses may initiate the respiratory infection, secondary bacterial infection may occur and simply identifying a virus at presentation (leading to antibiotics being with-held) may not indicate the sole aetiology of the child’s acute clinical presentation. This is exemplified on a large-scale in previous influenza epidemics where deaths were caused by secondary bacterial pneumonia rather than the influenza infection [16,57].

A systematic review of studies published between 1975 and 1999 that examined the radiological differentiation between bacterial and viral lower respiratory infection of children less than 18 years of age concluded that the degree of accuracy of the CXR was not clinically useful [58]. Only five studies met the inclusion criteria for this review. Over a decade later, little has changed [12,59]. The addition of other diagnostic markers such as cross-reactive protein (CRP), serum procalcitonin and interleukin-6 concentrations does not substantially improve aetiological differentiation [60–62].

The relative contribution of bacteria to lobar consolidation and the spectrum of CXR changes representative of severe pneumonia [24,25] varies considerably. Studies using lung aspirates reported bacterial causes between 28% and 82% of lobar or bronchopneumonia, with between 1% and 54% of these being *S. pneumoniae* [26].

The contribution of bacteria to interstitial infiltrates on CXRs is less certain. Overall, the precision of the term “infiltrate” as a diagnostic predictor in clinical settings is low [63]. In one study, 54% of 151 physicians surveyed thought infiltrate could mean any of six or more different pathophysiologic conditions, including nonspecific pneumonia, interstitial pneumonia, viral pneumonia, consolidation, or nonspecific interstitial process [63]. A prospective Finnish study using multiple bacteriological and virological methods to obtain a diagnosis reported a 50% split between viral and bacterial causes in children (*n* = 77) with interstitial infiltrates only [64].

### 2.4 CXR diagnosis of childhood pneumonia in the clinical context

The use of CXRs in the diagnosis of pneumonia should be limited to children with clinical signs suggesting severe pneumonia who require hospitalisation given there is no strong evidence to support its role in ambulatory settings even if the clinical findings in the child strongly indicate pneumonia [22,23]. However, it may be indicated in children with prolonged fever and cough, including children without tachypnoea and respiratory distress [22]. In children requiring hospitalisation, a CXR is indicated in the presence of signs suggesting severe pneumonia (hypoxia, tachypnoea, grunting, chest indrawing and/or crackles on auscultation), particularly in the presence of fever [65]. Lateral CXRs are not useful or necessary [66], unless confirmation of the presence of pleural fluid is required. A CXR beyond the initial procedure on admission is rarely necessary (see below).

#### 2.4.1. CXR contribution to the management of paediatric pneumonia

There is limited evidence to support the role of the CXR in the ongoing management of paediatric pneumonia in the absence of immune compromise, complications and/or failure to improve [67], and none that demonstrate that it positively influences clinical outcomes [68,69]. In an analysis of 100,615 presentations for community-acquired pneumonia (CAP) to 36 paediatric EDs in the United States, centres that employed more diagnostic tests (e.g. CXR, blood culture and complete blood counts) had a higher odds ratio (OR) of children being hospitalised than low testing centres (OR 1.86, 95% CI 1.17–2.94), but there was no significant difference in revisit rates (OR 1.21, 95% CI 0.97–1.51; *p* = 0.09) [70]. In a study of hospitalised children with CAP, increased utilisation of diagnostic testing was associated with longer lengths of stay (*p* = 0.036) but not with the probability of readmission (*p* = 0.225) [70]. However, in severe pneumonia, a CXR with ‘significant pathology’ (defined by WHO criteria [26]) has been associated with a high risk of antibiotic treatment failure [71], particularly penicillin compared to amoxicillin.

The CXR also has a limited role in the follow-up of children post-discharge unless cough persists and/or other signs suggest the child has not completely recovered [72,73]. Exceptions may include those with lobar collapse and recurrent pneumonia affecting the same lobe [22], or children with recurrent pneumonia where slow resolution of CXR changes in children hospitalised with pneumonia predicts 12-month chronic respiratory disease during the following 12 months [74].

### 2.5 CXR diagnosis of childhood pneumonia in the research context

Determining the burden of disease due to paediatric pneumonia, and evaluating interventions, has long been complicated by the lack of a reliable, standardised case definition for clinical and radiologically confirmed pneumonia that can be applied within and between studies. While CXR confirmation of pneumonia is considered the gold standard [75], its applicability in research has been complicated by wide inter- and intra-observer variability.

The development of vaccines and associated clinical trials for bacterial infections due to Hib and *S. pneumoniae* highlighted the difficulties associated with the lack of a standardised and valid case definition. A validated case definition was necessary for the provision of reliable measurement of vaccine efficacy for pneumonia, particularly non-bacteraemic pneumonia. In the mid 1990s, several research groups around the world met periodically with the WHO to discuss common methodological, design and logistic issues. By 1999, an objective of the group (referred to as the WHO Pneumonia Vaccine Trial Investigators Group) was to define the criteria for radiologically diagnosed pneumonia in children for the purposes of vaccine trial and burden of disease endpoints, particularly trials of pneumococcal and Hib conjugate vaccines. This process eventually led to the 2001 publication of the WHO protocol, *Standardization of interpretation of chest radiographs for the diagnosis of pneumonia in children* [26].

The group reviewed existing data from aetiological studies comparing CXR changes with isolates obtained from lung aspirates together with data and films from burden of disease studies in various countries. Over several meetings, the group arrived at a consensus definition of bacterial pneumonia. For the purposes of epidemiological studies, radiologically diagnosed pneumonia endpoints were defined as:

1. ***Significant pathology:*** this refers specifically to the presence of consolidation, infiltrates or effusion. If none of these are present then no further reading or recording is required for that film.
2. ***End-point consolidation:*** a dense opacity that may be a fluffy consolidation of a portion or whole of a lobe or of the entire lung, often containing air bronchograms and sometimes associated with pleural effusion.1
3. ***Other (non-end-point) infiltrate:*** linear and patchy densities (interstitial infiltrate) in a lacy pattern involving both lungs, featuring peribronchial thickening and multiple areas of atelectasis. Lung inflation is normal to increased. It also includes minor patchy infiltrates that are not of sufficient magnitude to constitute primary end-point consolidation, and small areas of atelectasis which in children can be difficult to distinguish from consolidation.
4. ***Pleural effusion:*** this refers to the presence of fluid in the pleural space between the lung and chest wall. In most cases this will be seen at the costo-phrenic angle or as a layer of fluid adjacent to the lateral chest wall. This does not include fluid seen in the horizontal or oblique fissures. Pleural effusion is considered as primary end-point if it is in the lateral pleural space (and not just in the minor or oblique fissure) and is spatially associated with a pulmonary parenchymal infiltrate (including other infiltrate) OR if the effusion obliterates enough of the hemithorax to obscure an opacity [26].

The protocol requires CXRs to be collected, scanned, and read in a systematic way and specifies the criteria for assigning a diagnosis of pneumonia. Two independent personnel (preferably a paediatrician and a radiologist) read each film, with discordant diagnoses reviewed by a separate expert panel.

The WHO compiled a set of 222 films for ongoing training, standardisation, calibration and quality control. An evaluation of the protocol [26] using these films was published in 2005 [50]. The study assessed intra- and inter-rater agreement among a group of 20 clinicians and radiologists. Reference readings used as the “gold standard” concluded that 43% of the 208 interpretable films indicated the presence of primary EPC. The proportion that individual readers classified as EPC ranged from 8% to 61%. The median sensitivity and specificity for clinician diagnosis of EPC compared with the reference reading was 0.84 and 0.89, respectively; for radiologists it was 0.87 and 0.87, respectively (ranges for the two groups not reported). The median kappa indices were 0.65 for clinicians and 0.73 for radiologists. Intra-observer agreement was high with a median of 88.5% (range: 76% to 99%) of films classified the same way on repeat testing. Overall a moderate level of agreement was achieved. The study indicated that the protocol, while imperfect could be implemented by study sites with the appropriate amount of training. A minimum standard for case ascertainment was available. Individual reader variability appears problematic; however, the protocol’s requirement for films to be read by two readers, with adjudication of discordant readings by an independent panel, is intended to minimise the impact this has on EPC estimates within studies.

The major questions posed by the WHO protocol [26] are a) how reliable is the method within and between researchers and various research populations, and b) how sensitive and specific is it for a diagnosis of bacterial pneumonia? While the protocol is now a major component of burden of disease and vaccine trial methodologies, few studies have reported their agreement between readers on the diagnosis of WHO defined consolidation on CXRs [43,50,76,77]; a limited number have reported the outcomes of calibrating readers to the WHO definition using their training images [51,78]. Furthermore, it appears there has been no minimum acceptable level of sensitivity, specificity or inter-observer agreement between readers established for these studies in either the training phases using the WHO set of 222 images or in the studies themselves [43,50,76,77].

The WHO protocol suggests sample size estimates for evaluating agreement be based on a kappa index of 0.8 and precision of ±0.1, and that a reasonable minimum value of both sensitivity and specificity of 0.8 [26]. The evaluation of the protocol interpreted a kappa index of >0.6 for 13/20 readers against the reference set as a reasonable level of agreement [50]. Fourteen of the 20 readers had sensitivities and specificities of ≥0.70 in identifying EPC. If this study is assumed to be the benchmark, then study sites using the protocol should be calibrated to at least these values.

In the retrospective review of radiographs from the Californian 7-valent pneumococcal conjugate vaccine trial [76], readings were conducted by a paediatric radiologist and a paediatrician; the panel for discordant films consisted of two radiologists. Of 521 films classified as ‘positive’ by either primary reader, the concordance rate for a positive reading by both readers was 48%. Amongst discordant films, the panel found 35% of reader A’s and 46% of reader B’s films were positive. This provided a set of 361 films considered as the standard for positive films. Against this set, the sensitivity and specificity of reader A was 82% and 97%, respectively. The corresponding values for reader B were 88% and 97%, respectively. The kappa value for the two readers was 0.58 (95% CI 0.54–0.63).

In a burden of disease study in Chile [77], a kappa coefficient of 0.58 was reported for agreement between a paediatrician and paediatric radiologist although no further details were provided. In a study of non-severe pneumonia in Pakistan [43], pneumonia was reported in 14% and 23% of 1,848 CRXs read independently by two radiologists; 23% of 371 discordant films were classified as positive by a third radiologist. The kappa statistic for the two initial readers was 0.46. A study of children presenting to emergency departments in Brazil with cough and tachypnoea in which 14.3% of films (*n* = 182) were positive reported a kappa of 0.70 (95% CI 0.56–0.83) for two radiologists [79]. It appears this study did not use a panel for discordant films.

These data suggest that agreement to date has, at best, been moderate and is likely to be dependent on the prevalence of the endpoint in different settings. They also clearly highlight the need for adequate training and an independent panel (also calibrated to the WHO definition) to review discordant films. Importantly, the lack of detail in most studies that have used the WHO protocol on the outcomes of calibration to the WHO definition needs to be addressed. Similarly, the usefulness of the protocol is dependent on film quality and, as such, ongoing quality control measures are critical [80].

A number of vaccine efficacy trials have now been conducted using the WHO protocol [76,81,82]. Vaccine trials have acted as probe studies to estimate the relative contribution of Hib and *S. pneumoniae* to lobar consolidation [83], and relatively consistent vaccine efficacy estimates ranging from 20% to 37% have been reported. However, eligibility criteria for entry into a vaccine trial limits the generalisability of their findings to the wider population, particularly given substantial differences in the epidemiology of these pneumonias within and between different populations. Similarly, most studies have used the WHO clinical definition for pneumonia as the indicator for the CXR and, as discussed previously above, there are limitations to this definition.

An Australian study used the WHO protocol to evaluate all CXRs taken in all hospitalisations for Indigenous children aged <5 years over a 10 year period, irrespective of inpatient diagnosis [84]. In this study, the CXR readers were blinded to the clinical history and to each other’s reading. Overall there were 24,115 hospitalised episodes of care for 9,492 children and 13,683 CXRs were taken within 3 days of admission. A CXR was obtained in 57% of all hospital admissions. WHO EPC was diagnosed in 11.6% of all episodes with CXR, and in 4.2% of these episodes there was no corresponding respiratory diagnosis. In addition, EPC has also been diagnosed in a relatively high proportion of cases of bronchiolitis, influenza and confirmed RSV infections in young infants [84,85], hence complicating the use of the WHO criteria in studies focusing on bacterial pneumonia during periods of high virus activity. Later studies have incorporated the use of CRP to improve the specificity of diagnosis, however its usefulnessvaries between populations [86–88].

### 2.6 Extending WHO EPC to clinical studies

**Figure 3:**
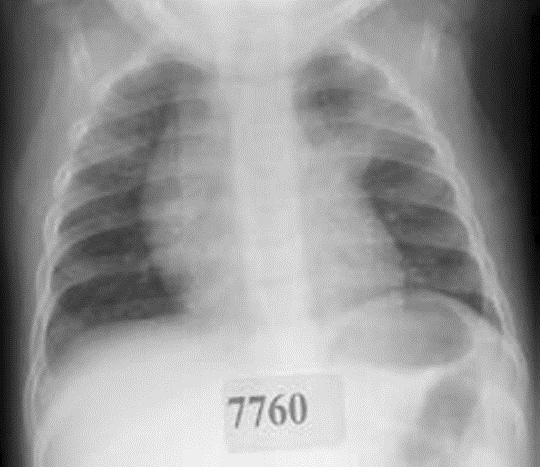
Example of chest radiograph discordant for the diagnosis of pneumonia between a paediatric pulmonologist and WHO radiological criteria when applied in the clinical context. Note: Chest radiograph of left upper lobe pneumonia diagnosed by a paediatric pulmonologist in a 12 month old child hospitalised for pneumonia. Clinical signs of tachypnoea, chest-indrawing and crackles on admission. This film was classified as negative according to WHO radiological criteria.

While the WHO radiological protocol [26] has its limitations, it currently remains the only tool for the standardisation of the radiological diagnosis of pneumonia in children. However, reports of studies that have used the protocol in clinical settings have emerged [71,89]. This is of some concern given the protocol is not intended for use in the clinical context. That the protocol underestimates alveolar pneumonia in particular is known [41,86], and may be partially attributed to the protocol’s subjective measure of CXR quality [41]. An Australian study examined the concordance between the radiological diagnosis of alveolar pneumonia using the WHO criteria to that of a paediatric pulmonologist (also calibrated to the WHO criteria) in Aboriginal children hospitalised with pneumonia and requiring antibiotics (Figure 3) [41]. Of the 147 episodes analysed, WHO-EPC was significantly less commonly diagnosed in 40 episodes (27.2%) compared to the paediatric pulmonologist’s diagnosis (difference 20.4%, 95% CI 9.6–31.2; *p* < 0.001). Clinical signs on admission were poor predictors for both the WHO and pulmonologist’s diagnoses; the sensitivities of clinical signs ranged from a high of 45% for tachypnoea to 5% for fever + tachypnoea + chest-indrawing. The positive predictive value (PPV) range was 40% to 20%, respectively. Higher PPVs were observed against the paediatric pulmonologist’s diagnosis compared to WHO-EPC. The paediatric pulmonologist’s heightened focus on the appearance of the right middle and left lower lobes that do not meet the WHO-EPC definition given the effect of the heart shadows/borders was considered an important influence. The WHO radiological guidelines also have limited value in the diagnosis of non-alveolar pneumonia, with one study demonstrating poor agreement between clinicians and radiologists (kappa = 0.23). This disagreement was associated with overdiagnosis by paediatricians, potentially leading to overtreatment [90].

In the clinical setting, a PPV is of greater importance than a negative predictive value to ensure children are adequately treated; the reverse is more often important in research settings (particularly vaccine efficacy and effectiveness studies) [91]. Clinical studies need to carefully assess the relevance of the protocol to the study population and the research objectives. Similarly, the processes required to use the WHO definition of radiological pneumonia (e.g. calibration to the WHO training films and the use of at least two blinded readers together with an independent panel for discordant films) need to be maintained.

## 3. Lung ultrasonography (LUS)

The initial LUS as a diagnostic tool in paediatric pneumonia with the identification of sonographic air bronchograms within lung consolidation, was published by Weinberg et al [92] in 1986. However, until recent advances in technology lead to the availability of portable, handheld ultrasonography (US) machines, its use has been largely confined to a supplemental role in evaluating complex pneumonia [93].

LUS has many potential advantages in the paediatric setting over CXR and CT scan including low-cost and simplicity, as well as the fact that ultrasound incurs no risk of radiation damage. While LUS is best performed by trained sonographers, medical students, doctors and other health care workers at the bedside are now being trained in it use, albeit cautiously [94,95]. This raises the potential for diagnostic capabilities in rural and remote settings where other imaging modalities are not available. Hence, in many clinical settings where rapid diagnosis can facilitate improved clinical outcomes and potentially reduce antibiotic use, interest in the use of LUS for the diagnosis of pneumonia in children is growing [96,97].

LUSs are performed using high-resolution linear probes with longitudinal and transverse sections of the anterior, lateral and posterior chest walls obtained [98]. The anterior section is defined by the chest between the parasternal and anterior axillary lines, the lateral section is between the anterior and posterior axillary line, and the posterior area is beyond the posterior axillary line [98]. Horizontal artifacts, defined as A-ines, reflect acoustic impedance at the pleura-lung interface and vertical artifacts, defined as B-lines, indicate interstitial or alveolar abnormalities that correlate with lung interstitial fluid content [99]. LUS signs of pneumonia include subpleural lung consolidation, B-lines, pleural line abnormalities, pleural effusion and the presence of sonographic air bronchograms [92,100]. B-lines, confluent B-lines or small areas of sub-pleural consolidations suggest viral pneumonias [101,102].

While a review of the evidence comparing the sensitivity and specificity of LUS to CXR for the diagnosis of CAP in adults identified several studies [103], and addressed consensus for the ongoing development and implementation of its use as point of care diagnostic tool [103], there are limited high quality studies in children in a range of populations. In the largest study to date, Shah et al [104] compared LUS to CXR in 200 children and young adults (median age 3 years) presenting to an ED in New York with suspected CAP. Both LUS and CXR were performed on all children, with the reference standard being the attending paediatric radiologist’s reading of the CXR. Inter-observer agreement with respect to radiologist readings of CXRs was not reported. Study sonologists were ED physicians with varying levels of US experience who underwent a short training program before the study started. Sonologists were reportedly blinded to CXR results and radiologists were blinded to LUS findings. Pneumonia was considered present on LUS if lung consolidation (defined as a subpleural echo-poor or tissue-like region with blurred margins or wedge shaped borders) with sonographic air bronchograms was visualised. For the diagnosis of pneumonia, LUS had an overall sensitivity of 86% (95% CI 71%–94%), specificity of 89% (95% CI 83%–93%), positive likelihood ratio of 7.8 (95% CI 5.0–12.4) and negative likelihood ratio of 0.2 (95% CI 0.1–0.4) [104].

Copetti et al [100] compared LUS with CXR in 79 Italian children aged 6 months to 16 years presenting to ED with suspected pneumonia. In these children, 60 children had positive LUS findings and 53 children had positive CXRs; there were no children who were CXR positive and lung ultrasound negative. Of the seven children who had a negative CXR but positive LUS, pneumonia was confirmed in four cases by thoracic CT and the authors reported the clinical course was consistent with pneumonia in the remaining three [100]. Neutrophil counts were elevated in 53 children with positive LUS and CRP counts were elevated in all 60 children with positive LUS.

While the studies above are promising, there are substantial limitations to both designs that necessitate caution, particularly the lack of any indication of the degree of agreement between radiologists with respect to CXR readings. Similarly, there is insufficient information with respect to the duration and severity of the illness at the time of presentation, relatively wide age ranges and insufficient eligibility criteria that would have excluded children with other important comorbidities that may have influenced clinical presentation and CXR findings at the time.

Further, anatomically the LUS would not be able to detect many regions of the lung using current US techniques. Segments that will invariably be undetectable by US are the medial segments of the lung distant to the chest wall which may be obscured by intervening aerated lung. Hence, there remains insufficient evidence to validly use LUS as both a diagnostic and management tool for uncomplicated paediatric pneumonia, although it does play an important role as a second line approach to confirm the diagnosis of empyema in children [96]. Furthermore, there are as yet no studies that have attempted to determine whether LUS can differentiate between viral and bacterial infections with sufficient specificity to inform clinical management. Similarly, there are no studies that have determined the effectiveness of LUS in the ongoing management of pneumonia over the course of an illness.

## 4. Computed Tomography (CT)

Predictably, adult studies have shown that CTs are significantly more sensitive than CXRs in the diagnosis of pneumonia [105], but the role of CT in paediatric pneumonia is still evolving. The use of CT in the diagnosis of pneumonia in children is usually limited to tertiary settings, predominantly in developed countries, given the resources required to perform the scan and the expertise needed to interpret the images. Even in those settings, the role of CT is generally confined to complicated pneumonias, particularly where parapneumonic effusion and empyema is suspected but not confirmed on CXR/LUS [22,106,107], in children who are immunocompromised [107] and to identify an underlying cause of pneumonia such as a foreign body [108] or sequestration [109]. CT may also be used to guide lung biopsy in cases where a specific aetiological diagnosis is required [110].

While CT is more sensitive in detecting parenchymal abnormalities than CXR [38,111,112], there is limited evidence to indicate the test alters management or that it can predict clinical outcomes [107,113]. There is also limited evidence to support the role of CT in reliably differentiating between viral and bacterial infections given the occurrence of overlapping features [114]. Other non-infectious conditions such as acute eosinophilic pneumonia and pulmonary haemorrhage can mimic bacterial pneumonia on CT [115]. CT also has many disadvantages in paediatrics. It requires sedation or anaesthesia in an uncooperative child and the dose of ionising radiation required is much higher than CXR. Radiation dose reduction is of particular importance in children as they are more susceptible to the risks of radiation [116].

## 5. Magnetic Resonance Imaging (MRI)

MRI of the lung provides both morphological and functional information and is an attractive non-radiation alternative in paediatrics [117]. In pulmonary infections involving alveolar infiltration or exudate patterns, it is thought that MRI can reliably depict these patterns and that the images are clearer than CXRs in segmental pneumonia and bronchopneumonia [118]. The role of MRI in diagnosing interstitial infections offers no advantage over the CXR [118]. However, access to MRI facilities is even more limited than that of CT. Also, a significant proportion of very young children will develop dorsal atelectasis associated with sedation during MRI and this may mask pathological processes [119].

Despite its potential advantages, there are limited studies that have investigated the role of MRI in paediatric pneumonia. In one small case series of 24 Turkish children with suspected lung infection, uncomplicated CAP was diagnosed in ten children [120]. Alveolar or interstitial parenchymal changes were detected in all acute cases and enlarged enhancing lymph nodes were seen in the hilar, mediastinal and axillary regions in the majority of children. In another study comparing the efficacy of chest MRI with fast imaging sequences to CXR in 40 children with pneumonia [121], all consolidation, lung necrosis/abscess, bronchiectasis, and pleural effusion detected with CXRs were also detected with MRI. There was a high level of agreement between CXRs and MRI in detecting consolidation (kappa = 0.78) in children with pneumonia. The agreement between CXRs and MRI was moderate for detecting pleural effusion (kappa = 0.30). While the small amount of available data suggests MRI can be a reliable alternative to the CXR, there remains insufficient evidence to support its role in determining aetiology, informing ongoing management or predicting clinical outcomes.

## 6. Conclusion

Radiology is widely used as an important, albeit imperfect, clinical tool in the diagnosis of paediatric pneumonia in some settings. However, like the many knowledge gaps in the management of childhood pneumonia [11], questions on radiology applications remain. As with most diagnostic approaches, its utility is dependent on the setting, the clinical presentation of the child, the experience of the clinician, radiographer and radiologist and the epidemiology of disease in the source population. The CXR is the most widely used approach but has limited value in mild illness, in predicting clinical outcomes and in differentiating between viral and bacterial infection. Other modalities such as LUS, CT and MRI have been proposed but their practical utility are currently questionable in noncomplicated disease.

In the research context, a standardised method for the interpretation of CXRs in studies of bacterial pneumonia in young children exists. While it has played an important role in vaccine trials, effectiveness studies and burden of disease research targeting *S. pneumoniae* and Hib, this tool designed for epidemiological purposes should not be extrapolated for clinical use [41,90]. Further, improvements in both the sensitivity and specificity of the WHO definition for radiologically confirmed pneumonia are needed. Considerable effort needs to be directed at achieving high inter- and intra-rater agreement and radiograph quality if it is to be a reliable definition for pneumonia in research.

There are considerable gaps in knowledge with respect to the radiological diagnosis of pneumonia in children [11], particularly in determining aetiology. Studies that have been conducted often have limited generalisability to other settings given variations in the epidemiology of disease worldwide. Large population-based studies are needed but are resource intensive and may be ethically questionable given radiation exposure, particularly in ambulatory settings. Advanced molecular methods may be an important contribution to the field, however improvements in the radiological diagnosis must also be accompanied by similar improvements in the clinical diagnosis of paediatric disease.
